# New insights into the unfolded protein response in stem cells

**DOI:** 10.18632/oncotarget.9833

**Published:** 2016-06-06

**Authors:** Yanzhou Yang, Hoi Hung Cheung, JiaJie Tu, Kai Kei Miu, Wai Yee Chan

**Affiliations:** ^1^ Key Laboratory of Fertility Preservation and Maintenance, Ministry of Education, Key Laboratory of Reproduction and Genetics in Ningxia, Department of Histology and Embryology, Ningxia Medical University, Yinchuan, Ningxia, P.R. China; ^2^ The Chinese University of Hong Kong–Shandong University Joint Laboratory on Reproductive Genetics, School of Biomedical Sciences, Faculty of Medicine, The Chinese University of Hong Kong, HKSAR, China

**Keywords:** endoplasmic reticulum stress, unfolded protein response, stem cells, differentiation, self-renewal

## Abstract

The unfolded protein response (UPR) is an evolutionarily conserved adaptive mechanism to increase cell survival under endoplasmic reticulum (ER) stress conditions. The UPR is critical for maintaining cell homeostasis under physiological and pathological conditions. The vital functions of the UPR in development, metabolism and immunity have been demonstrated in several cell types. UPR dysfunction activates a variety of pathologies, including cancer, inflammation, neurodegenerative disease, metabolic disease and immune disease. Stem cells with the special ability to self-renew and differentiate into various somatic cells have been demonstrated to be present in multiple tissues. These cells are involved in development, tissue renewal and certain disease processes. Although the role and regulation of the UPR in somatic cells has been widely reported, the function of the UPR in stem cells is not fully known, and the roles and functions of the UPR are dependent on the stem cell type. Therefore, in this article, the potential significances of the UPR in stem cells, including embryonic stem cells, tissue stem cells, cancer stem cells and induced pluripotent cells, are comprehensively reviewed. This review aims to provide novel insights regarding the mechanisms associated with stem cell differentiation and cancer pathology.

## INTRODUCTION

ER stress is triggered by a disturbance in endoplasmic reticulum homeostasis as a result of Ca^2+^ depletion, hypoxia, or N-terminal glycosylation dysfunction [1, 2]. The misfolded and unfolded proteins accumulate in the ER during stress, resulting in initiation of the unfolded protein response (UPR) to alleviate the side effects from ER stress and promote cell survival [3-5]. Therefore, the UPR is an evolutionarily conserved adaptive mechanism for increasing cell survival under ER stress conditions [6, 7].

Apoptosis is induced by persistent or excessive ER stress *via* the activation of the following three ER stress-mediated apoptotic pathways: (1) pro-apoptotic molecular CHOP (C/EBP-homologous protein, growth arrest and DNA damage-inducible gene 153[GADD^153^] and DNA—damage inducible transcription 3[DDIT^3^]); (2) phosphorylated c-Jun N-terminal kinase (p-JNK); and (3) cleaved caspase-4 in humans and caspase-12 in rodents [8-16].

The UPR is initiated to relieve the ER load through the following three pathways: (1) PERK (pancreatic ER kinase)/eIF2α (eukaryotic initiation factor 2α)/ATF4 (activating transcription factor 4); (2) IRElα (inositol requiring enzyme 1α)/XBP-1 (X-box-binding protein); and (3) ATF6α (activating transcription factor 6α). It is accompanied by the dislocation of the ER chaperonin glucose-regulated protein 78-kDa (GRP78, also known as Bip) from the ER membrane with PERK, IRElα, and ATF6α; from there, GRP78 enters the ER lumen [8]. Through these three pathways, the ER load is ameliorated by following three methods: (1) a reduction in the entry of newly synthesized proteins into the ER through attenuating protein translation; (2) an increase in the protein-folding capacity by upregulating ER gene expression; and (3) the degradation of misfolded and unfolded proteins through ER-associated degradation (ERAD) and lysosome-mediated autophagy.

The misfolded and unfolded proteins are mainly degraded by ERAD through the ubiquitin-proteasome system (termed ERAD I) [17, 18], though lysosome-mediated autophagy is also triggered when the ERAD is impaired, therefore, lysosome-mediated autophagy has been referred to as the ERAD II pathway [17, 19].

The role of the ER stress and the UPR in several physiological and pathological processes has been previously reviewed. However, the comprehensive role of ER stress and the UPR in stem cells has not been summarized.

Stem cells have been identified in various tissues. These cells correlate with development, tissue renewal and some disease processes. Many stem cells persist throughout the entire adult life of the organism [20]. This observation raises questions about quality maintenance and cellular homeostasis mechanisms. Several papers have highlighted the importance of autophagy in stem cells [20-24] and the activation of autophagy in these cells during self-renewal, pluripotency, differentiation and quiescence [23, 24]. Consistent with autophagy, the UPR is also confirmed as an evolutionarily conserved adaptive mechanism to maintain cell homeostasis through protein synthesis, remolding and degradation, and crosstalk between autophagy and ER stress has been widely revealed in several studies [25]. ER stress mediates autophagy [26], whereas autophagy inhibits ER stress [27]. The relationship between autophagy and ER stress depends on the cell type and conditions. Oxidative stress, mitochondrial dysfunction and ER stress also interact with one another [28-31]. Moreover, the interplay among oxidative stress, mitochondrial dysfunction and autophagy is dependent on cell type [32-33]. Mitochondrial function and oxidative stress are all widely related to stem cells [34-37]. However, it is largely unknown whether ER stress and the UPR interact with mitochondrial dysfunction, oxidative stress and autophagy in stem cells.

Thus, in addition to autophagy, the vital role of ER stress and the UPR in stem cells should be emphasized.

## ER STRESS AND THE UPR IN EMBRYONIC STEM CELLS

Embryonic stem cells (ESCs) are derived from blastocyst the inner cell mass (ICM). *In vivo*, they differentiate into the three primary germ layers: the ectoderm, endoderm and mesoderm [38]. These differentiation processes are regulated by complicated mechanisms.

### UPR in embryonic development

The development of a peri-implantation embryo is inhibited by excessive UPR. Following embryo demise, the UPR is induced by ER stress mediated apoptosis, this situation can be reversed by the ER stress inhibitor TUDCA (tauroursodeoxycholic acid) [39-42]. Inhibiting ER stress signaling has yielded beneficial effects on embryo survival and long-term developmental potential [43]. Furthermore, blastocyst formation *in vitro* during preimplantation embryo development was prevented by UPR [44]. The role of ER stress and the UPR in preimplantation embryonic development and developmental arrest has been reviewed [7, 45]. Additionally, hypoxia supplies a ‘niches’ for embryonic stem and progenitor cells, and low oxygen (O_2_) regulates embryonic stem and progenitor cell differentiation [46]. Up-regulation of the UPR after hypoxia suggests potential roles for the UPR in embryonic stem and progenitor cells [47]. Heavy proteins loaded on the ER are comprised of metabolic and secreted enzymes, antibodies, serum proteins and extracellular matrix (ECM) components during development in different cell types. In these conditions, cellular homeostasis is restored by the UPR, which is initiated by physiological ER stress [48]. Early embryogenesis in IVP (*in vitro*-produced) bovine embryos depends on an appropriate balance between autophagy and ER stress [49]. Therefore, the UPR is involved in embryonic development and embryonic stem cell and progenitor cells. However, whether autophagy and ER stress correlate with each other in embryogenesis is unknown.

Embryo development failure is the result of a deficiency in several UPR-regulating genes. Targeted mutation of the mouse GRP94 (glucose regulated protein 94) gene disrupts development and perturbs ER stress and the UPR signaling pathway [50]. A activation of UPR-regulating genes and apoptotic cell death by deletion of ERp44 (endoplasmic reticulum (ER) resident protein 44) in mice and zebrafish causes embryonic lethality at E9.5 to E12.5 and abnormal heart development [51].

As an ER molecular chaperone, GRP78 is essential for embryonic cell growth and pluripotent cell survival. A GRP78 deficiency results in ceased proliferation and a massive increase in apoptosis in the ICM [52]. Ubiquitous and high expression of the UPR regulating genes GRP78 and GRP94 occurs in late embryogenesis, followed by downregulation immediately after birth, indicating the important role of the UPR in embryogenesis [53]. The vital role of the UPR and two glucose regulated proteins (GRPs) GRP78 and GRP94 in regulating homeostasis of organs originating from the endoderm, mesoderm and ectoderm has been reviewed [54].

### UPR in differentiation and pluripotency of ESCs

Embryonic stem cells are useful tools for studying the regulatory mechanisms of cell proliferation and differentiation. Induction of the UPR during neuronal differentiations of rBMSCs (rat bone marrow stromal cells) and mESs (mouse embryonic stem cells) as well as neuronal marker NF-L (neurofilament-L) expression by non-cytotoxic concentrations of ER stress inducers strongly suggest the potential role of the UPR in neuronal differentiation [55]. Indeed, ER stress participates in ESC differentiation induced by retinoic acid through ERAD (ER-associated degradation) [56]. ER stress-induced UPR enhances definitive endodermal differentiation and inhibits other germ layer commitments of ESCs in EBs (embryoid bodies) of mouse origin *via* both the Smad2 and β-catenin signaling pathways [57]. DKK3 (dickkopf homolog 3) regulates ESC-SMC (smooth muscle cell) differentiation by activating ATF6 and promoting myocardin expression [58]. ESC differentiation is ceased by blocking the ER stress-induced UPR, which has been shown in several studies. Although the undifferentiated condition are maintained by the addition of LIF (leukemia inhibitor factor) into the culture medium, the cells have the potential to differentiate toward meso-endoderm lineages in the long-term mESC cultures because of spontaneously secreted vascular endothelial growth factors (VEGFs), which are attributed to hypoxia-inducible factor alpha (HIF1α) and the ER stress induced-UPR. Therefore, blocking ER stress following the inhibition of the VEGF and VEGFR signaling pathways prevents cell differentiation and promotes mESCs self-renewal [59]. Inhibition of ER stress by TUDCA prevented the differentiation of ESCs into definitive endodermal cells even with activin A treatment [57].

The self-renewal of stem cells is maintained by a balance between the positive regulation pathway of promoting growth and the negative regulation pathway that promotes pro-apoptosis [60].

Blanco-Gelaz reported that hESCs must resolve ER stress in culture conditions to survive and maintain pluripotency [61]. ERp44 (endoplasmic reticulum protein 44)-deficient ESCs showed higher [Ca^2+^] transient amplitudes, elevated ROS, and enhanced sensitivity to Tu (tunicamycin, 5 μg/mL), which induced ER stress mediated apoptosis [51].

In summary, the UPR is involved in the proliferation, differentiation, self-renewal and ER stress mediated apoptosis of embryonic stem cells. Moreover, embryonic demise is caused by UPR dysfunction.

## HEMATOPOIETIC STEM/PROGENITOR CELLS AND THE UPR

Hematopoietic stem cells (HSCs) are widely studied because of the relative ease of access to cell populations in the bone marrow and blood for *in vitro* and *in vivo* assays. A large number of mature blood cell lineages are produced each day through the proliferation and differentiation of progenitors originating from a small number of quiescent stem cells. The life span of mature blood cells is very limited despite the fact that HSCs exist in the organs throughout life. HSCs are identified and isolated by the positive expression of cell surface protein markers (ATXN1/Sca-1 and KIT/c-Kit) or the negative expression of lineage markers (ITGAM/MAC-1 for mature myeloid cells, CD8 for T cells, and PTPRC/B220 for B cells) on immature cells [20, 21]. HSCs exist in hypoxic microenvironments (niche) in the bone marrow, and the majority of HSCs are in a quiescent state [62].

### UPR in the HSCs self-renewal

The blood system is maintained by a pool of HSCs that are long-living due to their capacity for self-renewal. Consequently, cell damage occurs by stress stimuli including reactive oxygen species (ROS), nutrient fluctuation and DNA damage [63, 64]. It is unclear how to prevent the loss of function and clonal persistence of oncogenic mutations that increase the risk of leukemogenesis in stressed HSCs [65, 66]. In the physiological stem cell hierarchy, the UPR has been shown to regulate the self-renewal capacities of HSCs, the importance of the UPR in HSCs has been reviewed by Kharabi Masouleh et al [67]. A gene expression analysis revealed that, PERK is predominately activated in HSCs compared to downstream progenitor populations [68]. The pharmacological PERK inhibitor GSK2606414 causes apoptosis in HSC populations, whereas the progenitor populations are resistant, even at high concentrations [68]. The UPR governs the integrity of the hematopoietic stem-cell pool during stress. Van Galen suggested that the HSC pool maintains clonal integrity by the clearance of individual HSCs after stress to prevent the propagation of damaged stem cells through the UPR to either resolve stress or initiate apoptosis [68]. Indeed, the UPR is induced by ROS [69], and ROS is initiated by ER stress [70].

### UPR in HSC death and homeostasis

Elevated ER stress and activation of the UPR are caused by a deficiency in UFBP1 (Ufm1 binding protein 1, also known as DDRGK1, Dashurin and C20orf116), which subsequently leads to the death of hematopoietic stem/progenitor cells. Thus, UFBP1 plays pleiotropic roles in the regulation of hematopoietic cell survival and differentiation *via* modulating ER homeostasis and erythroid lineage-specific gene expression [71]. A Runx1 deficiency of in hematopoietic stem and progenitor cells (HSPCs) results in slow growth, low biosynthesis, small cell phenotype and markedly reduced ribosome biogenesis (Ribi), which is consistent with lower p53 levels, reduced apoptosis, attenuated UPR, and resistance to genotoxic and ER stress [72]. Cripto regulates hematopoietic stem cells as a hypoxic-niche-related factor through the cell surface receptor GRP78 [73]. Soluble Cripto-1 has been shown to selectively regulate cells expressing high levels of surface GRP78, to activate the PI3K/Akt pathway and to promote stem cell maintenance ex vivo [74]. A proteomics analysis revealed that Cripto-GRP78 binding stimulates glycolytic metabolism-related proteins and results in lower HSC mitochondrial potential [74]. ER stress also correlates with mitochondrial bioenergetics [75, 76]. Cripto-GRP78 is a novel HSC regulatory signaling pathway that primarily works in the hypoxic niche [77]. Acute inducible ablation revealed a role for GRP78 in hematopoietic stem cell survival, lymphogenesis and stress signaling regulation [78]. Dppa5 overexpression robustly increases the reconstitution capacity of HSCs after *in vitro* culture, and ER stress levels and subsequent apoptotic signals are significantly decreased upon ectopic Dppa5 expression. Conversely, downregulation of Dppa5 expression abolished long-term reconstitution by HSCs because of increased apoptosis caused by elevated ER stress levels [79]. HIF2α protects human hematopoietic stem/progenitors and acute myeloid leukemia cells from apoptosis induced by ER stress [80]. RCAD/Ufl1, an Ufm1 E3 ligase, is essential for embryonic development, HSC survival and erythroid differentiation. The loss of RCAD/Ufl1 blocked autophagic degradation, increased mitochondrial mass and reactive oxygen species, and led to a DNA damage response, p53 activation and enhanced cell death of HSCs [81], accompanied by elevated ER stress and activation of the UPR [81]. ATF4-deficient mice showed fewer hematopoietic progenitor cells in the liver [82]. ATF4 is a downstream target of PERK, which is one pathway that imitates the UPR [7]. In contrast, myeloid cell and eosinophils progenitors selectively activate the endoribonuclease IRE1α and Xbp1 mRNA splicing for the UPR without inducing parallel ER stress signaling pathways [83]. Inherited heterozygous point mutations in the ELANE gene, which encodes neutrophil elastase (NE), activates severe congenital neutropenia (SCN) [84, 85]. Genome editing and differentiation protocol results obtained in studies with both normal and SCN patient-derived induced pluripotent stem cells (iPSCs) suggest that promyelocyte death and differentiation arrest are caused by ELANE point mutations and are associated with NE mislocalization and activation of the UPR/ER stress. This disadvantage is reversed by sivelestat, an NE-specific small-molecule inhibitor, and the UPR/ER stress is ameliorated [86].

### Mitochondrial UPR and HSC aging

The mitochondrial UPR is triggered by mitochondrial dysfunction or the accumulation of unfolded proteins within mitochondria, it promotes cell survival along with the repair and recovery of defective mitochondria [87]. The degeneration of mitochondrial quality and activity contributes to aging, including cellular senescence, chronic inflammation, and the age-dependent decline in stem cell activity [88].

Reduced quiescence in HSCs is caused by SIRT7 inactivation, accompanied by increases in the mitochondrial UPR (UPR^mt^) and compromised regenerative HSC capacity. SIRT7 up-regulation improved the regenerative capacity of aged HSCs *via* decreasing the UPR^mt^ pathway [34]. HSCs repress mitochondrial biogenesis and OxPhos (oxidative phosphorylation) *via* the UPR^mt^ signaling pathway to coordinate the metabolism required for stem cell maintenance [34].

In summary, the UPR maintains HSC self-renewal, while the ER stress mediated apoptotic pathway initiates HSC deaths. Whether HSC differentiation and proliferation are regulated by the UPR is unknown and must be explored in the future.

## NEURONAL STEM CELLS AND THE UPR

Recent studies have suggested that the ER stress induced UPR pathway is involved in neurodegenerative diseases, and that ER stress is a potential therapeutic target for preventing these diseases [89-92]. In addition to being involved in the pathology of neurological diseases, the UPR also plays vital roles in neural differentiation during brain development [53, 93-95]. All of these results emphasize the importance of ER stress and the UPR on neurogenesis.

### UPR in NSCs differentiation

ER stress causes aberrant neuronal differentiation of NSCs followed by neurite growth inhibition [96]. Induction of the UPR during rBMSC (rat bone marrow mesenchymal stem cell) and mESC (mouse embryonic stem cell) neuronal differentiation as well as NF-L (neurofilament) expression by ER stress inducers strongly suggest a potential role for the UPR in neuronal differentiation [54]. Human pluripotent stem cell (hCN)-derived cortical neurons (CNs) under mild-to-moderate hypothermia (28-32°C) induce cold-shock protein expression and mild ER stress, which subsequently activates the UPR. Blocking a principal UPR pathway mitigates the protective effect of cooling against oxidative stress, while pre-cooling neurons abrogates toxic injury produced by the ER stressor tunicamycin. Therefore, hypothermia is a potent neuroprotective mechanism that upregulates the adaptive chaperone-driven pathways of the UPR and even maintains ER -hormesis [97].

### UPR in NSCs self-renewal

In addition to resulting in neuronal cell death through calcium toxicity and apoptotic pathways, ER stress also triggers a series of adaptive responses including the UPR, autophagy, the expression of pro-survival proteins and the enhancement of ER self-repair abilities, leading to less ischemic brain damage [98].

Inhibition or down-regulation of DOT1L in neural stem cells (NSCs) in the cerebral cortex impairs NSC proliferation and survival, induces genes that are activated during the UPR in the endoplasmic reticulum (ER),the loss of H3K79me2 at the ATF4- and CHOP-promoters, which appears to mark a point-of-no-return that activates the death program in NSCs [99]. Activationgalanin receptor (GalR)3 increases NSC viability; this protective effect occurs because of decreased apoptosis and CHOP levels [100]. ER stress, mitochondrial dysfunction and calpain/JNK activation are involved in oligodendrocyte precursor cell death by unconjugated bilirubin [101]. Human cytomegalovirus induces apoptosis in neural stem/progenitor cells derived from induced pluripotent stem cells by generating mitochondrial dysfunction and ER stress [102]. FTD (frontotemporal dementia) neurons differentiated from induced pluripotent stem cells (iPSCs) from individuals with FTD-associated MAPT mutations show activation of the UPR; a transcriptome analysis of these cells exhibited disease-associated gene expression profiles [103]. Upregulation of GRP78 and GRP94 suggests that the USH2A variant (Arg4192His) in retinitis pigmentosa patients causes the disease through protein misfolding and ER stress [104]. These results indicate that the transcriptome is potentially reprogrammed by the UPR.

Therefore, the UPR is involved in neuron cell differentiation and self-renewal, and ER stress dysfunction initiates apoptosis.

## ESOPHAGEAL EPITHELIUM PRECURSOR / STEM CELLS AND THE UPR

The self-renewal of most epithelial tissues is attributed to the presence of stem cells, and the existence of esophageal epithelium precursor/stem cells has been demonstrated by several papers [105-109]. Esophageal epithelial stem cells demonstrate several unusual properties, and their identification may facilitate studies on esophageal carcinogenesis [106]. Controlling differentiation, proliferation and self-renewal is highly important to epithelium precursor/stem cells.

### UPR in the differentiation of esophageal epithelium stem/precursor cells

UPR induced epithelial differentiation in precursor cells in the esophageal epitheliummay serve as a quality control mechanism that protects against the development of esophageal cancer [110]. The expression of the highest and lowest levels of intestinal stem cell (ISC)-related genes in the CD44^hi^GRP78^−/lo^ and CD44^lo^GRP78^+^ cell populations reveals that the combination of GRP78 and CD44 can largely distinguish ISCs from progenitor cells [111]. Rapid loss of intestinal epithelial stem cells was caused by the initiation of the UPR by ER stress [112]. IRE1a acts as a novel mediator in regulating osteogenic differentiation through negative feedback with BMP2 [113].

### UPR in the self-renewal of esophageal epithelium stem/precursor cells

Intestinal stem cell proliferation is controlled by the integration of UPRER (the unfolded protein response of the endoplasmic reticulum) and the oxidative stress signaling pathway [114]. MSC proliferation is inhibited by treatment with low-intensity and intermittent negative pressure, accompanied by triggering ER stress-associated cellular apoptosis, which further enhances the osteogenesis activity and induces differentiation to osteoblasts [115]. The UPRER is a critical regulator of intestinal stem cell (ISC) quiescence in *Drosophila melanogaster*. Wang's report found that ISCs require activation of the UPRER for regenerative responses, though a tissue-wide increase in ER stress triggers ISC hyperproliferation and epithelial dysplasia in aging animals [114]. ADAR1 (adenosine deaminase acting on RNA 1) plays vital roles in survival, maintenance of intestinal stem cells and intestinal homoeostasis by suppressing ER stress and interferon (IFN) signaling [116]. The UPR transcription factor Xbp1 suppresses intestinal tumorigenesis by directly acting on intestinal stem cells [117]. PERK controls *Drosophila* lifespan by promoting ISCs proliferation in response to ER stress [118].

Hence, the UPR plays a vital role in esophageal epithelium stem/precursor cells processes, including differentiation, proliferation, self-renewal and quiescence.

## CANCER STEM CELLS AND THE UPR

Cancer stem cells (CSCs) are a small subset of cancer cells with the indefinite potential of self-renewal and the capability to drive tumorigenesis [119]. These cells are believed to be responsible for tumor initiation, progression, metastasis, chemotherapy and radiation resistance, and tumor relapse after therapy [120, 121]. Therefore, targeting CSCs is crucial for the effective treatment of cancer [122].

### UPR in CSC differentiation

Colon cancer stem cells (colon-CSCs) are more resistant to conventional chemotherapy than differentiated cancer cells [123-127]. The ER is a vital organelle, and that plays an important role in regulating the homeostasis of normal intestinal stem cells [128]. ER-stress-induced activation of the UPR forces colon-CSCs to differentiate [128], and the rapid loss of intestinal epithelial stemness results in their enhanced sensitivity to chemotherapy *in vitro* and *in vivo* [112]. Induced activation of the UPR may be used to increase the sensitivity of colon-CSCs to the effects of conventional chemotherapy [128]. Thus, the UPR plays an important role in the regulation of intestinal epithelial stem cell differentiation [128]. At the intestinal level, 17-allylamino-demethoxygeldanamycin (17AAG), which is an inhibitor of the stress protein HSP90, promotes splicing of the transcription factor X-box binding protein 1 (XBP1), accompanied by a decrease in intestinal damage and an increase in Lgr5+ stem cells [129]. Activation of the UPR in healthy intestines by genetic deletion of the ER chaperone GRP78 results in a loss of stem cells due to differentiation and a quick repopulation of healthy stem cells that were not recombined [112]. GRP78 as a pluripotent stemness regulator is up-regulated in oral cancer patients with an areca nut-chewing habit [130]. GRP78 may serve as a molecular target that can be further developed for the eradication of refractory HNC (head-neck cancer) with a stemness phenotype [131].

The antitumor effect of shikonin is enhanced by inhibiting endoplasmic reticulum stress *via* the JNK/c-Jun pathway in human glioblastoma stem cells [132]. Because SEL1L is an UPR and ER-associated degradation protein, downregulation of SEL1L sensitizes glioma stem cells to the cytotoxic effects of valproic acid [133]. *In vivo* RNAi screening for BMI1 targets suggests that the TGF-β/BMP-ER stress pathways are key regulators of neural- and malignant glioma-stem cell homeostasis [134]. Tumor cells that secrete GRP78 induce the differentiation of bone marrow mesenchymal stem cells to cancer-associated fibroblasts [135]. The metastases are caused by disseminated tumor cells (DTCs), which colonize secondary organs. Breast cancer DTC cell lines with stem/progenitor cell cancer phenotypes (CD44(high)/CD24(low)) show high expression of the UPR proteins GRP78 and GRP94. These observations provide novel insights for curing breast cancer by controlling the UPR [136].

### UPR in CSC self-renewal

High expression of Abcg1 in mouse and human LG-GSCs (low-grade glioma stem cells) is critical for protecting mouse LG-GSCs from ER-stress-induced apoptosis [137]. The major ER chaperone GRP78 is in a dynamic equilibrium between folding proteins and ER transmembrane receptors. Activation of the UPR is initiated by an increased load of folding proteins and accompanied by the dislocation of GRP78 away from the transmembrane receptors [128]. Cell surface levels of GRP78 are high in stem cells and tumor cells. The elevated Grp78 levels in human tumors are closely related with the promotion of tumor growth, malignancy and therapy resistance [138-140]. The dietary compound isoliquiritigenin (ISL) has synergistic effects with chemotherapeutic drugs for inhibiting breast cancer stem cell proliferation and colony formation, inhibiting self-renewal and multidifferentiation abilities, and targeting GRP78 *via* the β-catenin/ABCG2 pathway [141]. Ovarian cancer stem-like cells (ID8) increase the expression of plasma membrane GRP78 ((mem)GRP78), which increases self-renewing abilities. The down-regulation or inhibition of the GRP78 protein by antibodies from the carboxy-terminal domain of GRP78 reduces the self-renewal ability of ovarian cancer cells [142]. The mechanisms that allow residual multiple myeloma (MM) cells to persist after bortezomib (Bz) treatment remain unclear. A recent study suggested that GRP78 is expressed in MM quiescent cells. The expression of GRP78 was downregulated when a specific SubAB bacterial toxin was used to kill Bz-surviving MM cells. A high number of GRP78(high)/CD138+ MM cells are associated with progressive disease [143].

Pterostilbene (PT) treatment suppresses self-renewal and the irradiation-resistant abilities of GSCs (glioma stem cells) *via* negatively modulating the GRP78 signaling pathway. Subsequently, GSC development is suppressed [144]. GRP78 is required for cancer stem-like cell subpopulation in the MCF-7 breast cancer cell line that are resistant to IR (radiotherapy); knockdown of this gene augments the effects of IR, whereas GRP78 overexpression increases the radiation resistance of the subpopulation to IR [145]. BFA (Brefeldin A) effectively reduces the survival of suspension Colo 205 cells (IC50 = ~15 ng/mL) by inducing apoptosis and inhibiting the clonogenic activity of Colo 205 CSCs (cancer stem cells) in a tumor sphere formation assay and soft agar colony formation assay in the same nanogram per milliliter range. At such low concentrations, BFA effectively induced the UPR, which was indicated by the increased in the mRNA expression of UPR-related genes, such as GRP78, XBP1 and CHOP [146].

Overexpression of the REIC/Dkk-3 gene showed a potent selective therapeutic effect on various human cancers by triggering ER stress and the UPR [147].

Therefore, the importance of the UPR is demonstrated through its role in cancer stem cell self-renewal, differentiation, and apoptosis, which is dependent on the cell types. The roles of the UPR in cancer stem cells will provide a novel target for cancer therapy.

## MAMMARY STEM CELLS AND THE UPR

Mammary stem cells (MaSM) constitute a quiescent and self-renewing population isolated from fetal and adult mammary epithelial cells [148]. They are capable of developing into differentiated ductal, alveolar and myoepithelial cells [149].

The behavior of isolated stem cells from fetal and adult mammary epithelial cells is regulated by CRIPTO and its cell-surface receptor GRP78; CRIPTO inhibition promotes stem cell differentiation and reduces self-renewal when cultured ex vivo. In contrast, CRIPTO treatment maintains the stem cell phenotype and increases the mammary reconstitution capacity. Mammary gland reconstitution potential is ceased by the deletion of GRP78 from adult mammary epithelial cells. These results suggest that CRIPTO/GRP78 correlate with the fetal and adult mammary stem cell behavior ex vivo [150]. Furthermore, CRIPTO-1 activates branching morphogenesis and the epithelial-mesenchymal transition in the mammary gland both *in vitro* and *in vivo*, and together with the cofactor GRP78, it is critical for the maintenance of mammary stem cells ex vivo [151]. Knockout of GRP78 in mammary epithelial stem/progenitor cells fails to regenerate the mammary glands [152]. IGF-1 is involved in mammary gland development *via* GRP78 regulation [153].

In summary, the UPR is involved in the control of self-renewal, differentiation and multipotency in MaSCs.

## UPR IS INVOLVED IN THE DIFFERENTIATION OF ADIPOSE-DERIVED STEM CELLS

Adipose-derived stem cells (ASCs) are a population of multipotent stem cells that are isolated from the adipose tissue. They are capable of differentiating into various types of terminally differentiated cells, including but not limited to adipocytes, osteocytes and chondrocytes [154-158].

Tauroursodeoxycholic acid (TUDCA) treatment of hASCs significantly decreases the representative UPR regulating gene GRP78, the adipogenic markers peroxisome proliferator-activated receptor gamma (PPARγ) and glycerol-3-phosphate dehydrogenase 1 (GPDH), and lipid accumulation. Moreover, it decreases adipogenic differentiation from hASCs and significantly decreases *in vivo* adipogenic tissue formation and ER stress [159]. ER stress signaling reduces adipocyte differentiation and attenuates adipogenesis in mice by upregulating phosphorylated eIF2α and its downstream target CHOP but not IRE1α. ER stress inhibits adipocyte differentiation in 3T3-L1 cells [160]. In contrast, impaired eIF2α phosphorylation enhances adipocyte differentiation in mice MEFs (embryonic fibroblasts) [160]. Mice that are deficient for CHOP (CHOP^-/-^) gain more fat mass than wild-type mice on high-fat diets. Additionally, CHOP deletion in genetically obese Lepr^db/db^ (leptin receptor) mice increased body fat mass without altering adipocyte size. In contrast to the eIF2α-CHOP pathway, the activation or deletion of IRE1a (also known as Ern1) did not alter adipocyte differentiation in 3T3-L1 cells [160]. ER stress is involved in human adipocytes through the regulation of inflammation and adiponectin levels [161].

Thus, the UPR is involved in the differentiation of adipose-derived stem cells.

## UPR IS INVOLVED IN THE DIFFERENTIATION AND SELF-RENEWAL OF MSCS

Mesenchymal stem cells (MSCs) with self-renewal potential and multipotency can be isolated from various adult and fetal tissues. MSCs have the capacity to proliferate and differentiate into a variety of cell lineages such as osteoblasts, chondrocytes, adipocytes, fibroblasts, cardiomyocytes, etc. [162].

Palmitate induces apoptosis in hUC-MSCs (human umbilical cord-derived mesenchymal stem cells), which might be attributed to ER stress [163]. ER stress contributes to arsenic trioxide-induced intrinsic apoptosis in human umbilical and bone marrow mesenchymal stem cells; moreover, arsenic trioxide significantly reduces the viability of HUMSCs and HMSC-bm (bone marrow) in a concentration- and time-dependent manner [164]. LPA (lysophosphatidic acid) treatment decreases ER stress-mediated apoptosis in hypoxia and serum deprivation-stimulated mesenchymal stem cells [165]. Therefore, ER stress-associated apoptosis is involved in mesenchymal stem cell apoptosis [166-168]. GRP78 regulates ER stress-mediated apoptosis in cartilage development during the course of chondrocyte differentiation; GRP78 overexpression inhibits ER stress-mediated apoptosis, while GRP78 knockdown *via* siRNA activates the ER stress-specific caspase cascade in developing chondrocyte tissues from C3H10T1/2 cells [169]. Osteoblast proliferation and differentiation are impaired in Arl6ip5 knocked-down and deficient primary osteoblasts; it also induced ER stress and enhanced ER stress-mediated apoptosis. CHOP is involved in the regulation of apoptosis and differentiation in Arl6ip5 knocked-down osteoblasts [170] from the stromal/osteoblast cell line (UAMS-32). The cysteine-rich ER stress inducible factor with EGF-like domains 2 (Creld2) is an important mediator of BMP9-regulated osteogenic differentiation in mesenchymal stem cells [171].

IRE1a negatively regulates BMP2-induced osteoblast differentiation, and this IRE1a inhibition effect depends on the GEP (granulin-epithelin precursor) growth factor in pluripotent mesenchymal C2C12 cells [172]. Chondrocyte differentiation is controlled by IRE1α based on its enzymatic activity [173]. ATF6 positively regulates chondrocyte hypertrophy and endochondral bone formation through activating Runx2-mediated hypertrophic chondrocyte differentiation in C3H10T1/2 cells [174]. The chronic exposure of BMSCs (mesenchymal stem cells in bone marrow) to alcohol induces adipogenesis and disrupts or inhibits osteogenesis accompanied by inducing ER stress, ATF4/CHOP and activation of TNF-α signaling by ATF4/CHOP; the knockdown of either ATF4 and CHOP or TNF-α with siRNAs is able to reverse ethanol-induced adipogenesis [175].

Hence, the differentiation and self-renewal of MSCs is regulated by the UPR.

## CARDIAC STEM CELLS AND UPR

Cardiac stem cells have the capacity of self-renewal and can differentiate into multiple cell types [176, 177]; they are identified by the following markers: c-kit, Sca-1, and Isl1 [178].

PARM-1 (prostatic androgen repressed message-1) is an endoplasmic reticulum (ER) molecule that is involved in ER stress-induced apoptosis in cardiomyocytes.

PARM-1 plays an important role in the cardiomyogenic differentiation of P19CL6 cells by regulating the BMP/Smad signaling pathways, demonstrating a novel role for PARM-1 in the cardiomyogenic differentiation of stem cells [179]. This result hints at the potential role of the UPR in cardiac stem cells, but the accurate function of the UPR must be explored in the future.

## UPR AND IPSCS

Induced pluripotent stem cells (iPSCs) are self-renewable and can differentiate into different types of adult cells [180]. iPSCs are established through nuclear reprogramming by four key reprogramming gene factors (POU5F1(OCT4), SOX2, KLF4, and c-MYC) [181]; they are called the OKSM or Yamanaka factors [182]. The four factors have been thought to mainly modify gene expression profiles and epigenetic markings. Whether the UPR is involved in installing iPSCs remains unclear. A number of studies have revealed that the UPR is associated with reprogramming. Cell growth arrest and death are triggered by Gln (glutamine) and Glc (glucose) depletion, accompanied with dramatic gene expression reprogramming and distinguishable ER stress and the UPR [182]. Thus, the importance of the UPR in controlling transcriptional and translational reprogramming is reviewed [183]. Furthermore, c-myc translation and expression are regulated by ER stress inducers [184]. The UPR is activated during normal epidermal KC (keratinocyte) differentiation and induces C/EBPbeta, KLF4, and ABCA12 mRNAs [185]. Compared to protected sites, endothelia in athero-susceptible regions shows activation of ER stress and the UPR; moreover, there is evidence of altered expression of pro-inflammatory nuclear factor kappa B (NFκB) and oxidant/antioxidant pathways and low expression of major protective factors, notably endothelial nitric oxide synthase and Kruppel-like factors KLF2 and KLF4 [186]. Similar to ESCs, iPSCs show a reduction in mitochondrial mass and ROS generation [187, 188]. The adaptive UPR response affects mitochondrial function [189-191]. Additionally, ER stress and oxidative stress crosstalk with one another [192, 193]. These results suggest the potential role of the UPR in iPSCs.

## CONCLUDING REMARKS

**Figure 1:**
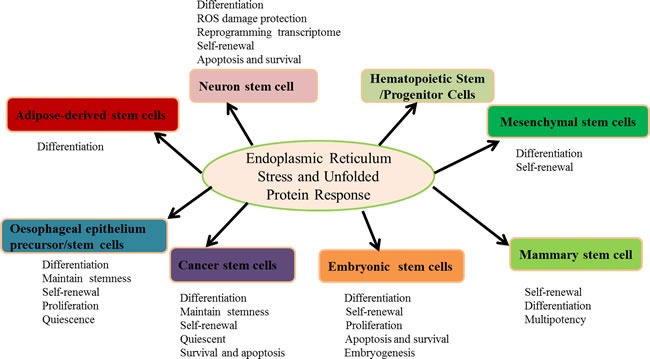
The roles of ER stress and the UPR in stem cells The activation of ER stress and the UPR are involved in differentiation, self-renewal, proliferation, survival and apoptosis to maintain multipotency. Furthermore, ER stress and the UPR govern the stem cell pool integrity and initiate adaptive responses to the hypoxic niche, regulate mitochondrial potential, and reprogram the transcriptome.

Stem cell self-renewal, pluripotency, differentiation and quiescence have been studied in the past decades, but knowledge of the role of ER stress and the UPR in stem cells is in its infancy. Stem cells are particularly vulnerable to stress. The UPR and ER stress have not yet been extensively studied in these cells; however, the data implicate pivotal roles for ER stress and the UPR in stem cells (Figure 1). A number of ER stress and UPR regulated genes are involved in stem cells (Table 1). Activation of the ER stress and the UPR is not only a mechanism for the eliminating of stem cells that encounter insults during development. Instead, activation of ER stress and the UPR might also be beneficial for directing stem cells into differentiation or for maintaining a proliferative status [96, 194, 195, 196]. Thus, the UPR must be tightly regulated and adapted to various cellular needs to balance cell fate decisions ranging from differentiation to cell death [99]. However, the role of ER stress and the UPR is dependent on the cell type. Therefore, (i) the functions of ER stress and UPR in the different stem cells should be explored further. (ii) The potential for controlling cell differentiation by regulating ER stress and the UPR should also be explored, particularly that of cancer stem cells. The latter could potentially provide novel insights for cancer therapy. (iii) Finally, the crosstalk between ER stress and autophagy, oxidative stress, and mitochondrial dysfunction should be explored to supply novel insights for controlling stem cell differentiation, proliferation, self-renewal and homeostasis maintenance.

**Table:** List of ER stress and UPR modulators in stem cell

Modulator	Functions	Reference
**ADAR1**	ADAR1 is essential for intestinal homeostasis and stem cell maintenance and survival through suppressing endoplasmic reticulum (ER) stress	[116]
**ASK1**	ASK1 knockdown in C17.2 neural stem cells diminished high glucose- or tunicamycin-induced IRE1α activation, reveals that ASK1 plays a causal role in diabetes- induced ER stress and apoptosis	[198]
**ATF6**	ATF6 upregulates XBP1s and inhibits ER stress-mediated apoptosis in osteoarthritis cartilage	[199]
**ATF6**	ATF6 positively regulates chondrocyte hypertrophy and endochondral bone formation through activating Runx2-mediated hypertrophic chondrocyte differentiation	[174]
**Bmi1**	In vivo RNAi screen for BMI1 targets reveals TGF-β/BMP-ER stress pathways as key regulators of neural- and malignant glioma-stem cell homeostasis	[134]
**BMP-2**	BMP-2 stimulates differentiation of myoblasts into osteoblasts through the PERK-eIF2α-ATF4 pathway, additionally stimulates Tmem119 and Tmem119 increases ATF4	[197]
**C/EBPβ**	Mechanical strain downregulates C/EBPβ in MSC as well as ameliorates Endoplasmic Reticulum Stress	[168]
**Creld2**	Endoplasmic reticulum (ER) stress inducible factor cysteine-rich with EGF-like domains 2 (Creld2) is an important mediator of BMP9-regulated osteogenic differentiation of mesenchymal stem cells	[171]
**DOT1L**	The lysine methyltransferase DOT1L/KMT4 promotes proliferation and protects cortical neural stem cells from activation of ATF4-DDIT3-mediated ER stress In Vitro	[99]
**Dppa5**	Dppa5 promotes hematopoietic stem cell activity by decreases endoplasmic reticulum stress	[79]
**ELANE**	Promyelocyte death and differentiation arrest are caused by ELANE point mutations, and is related with NE mislocalization and activation of the unfolded protein response/ER stress	[86]
**ERp44**	Deficiency of ERp44 in mouse and zebrafish results in significant embryonic lethality, abnormal heart development, altered Ca(2+) dynamics, reactive oxygen species generation, activated ER stress gene profiles, and apoptotic cell death	[51]
**GalR3**	GalR3 activation promotes adult neural stem cell survival due to decreased apoptosis and CHOP levels	[100]
**Grp78**	GRP78 is essential for the growth of embryonic cell and the survival of pluripotent cell	[52]
**GRP78**	GRP78 plays a pleiotropic role in BM cells and contributes to HSC survival and the maintenance of the lymphoid lineage through UPR signaling pathways	[78]
**HSP90**	Inhibition of the stress protein HSP90, promotes the splicing of the transcription factor X-box binding protein 1 (XBP1), accompanied with a decreasing intestinal damage and an increasing Lgr5+ stem cells	[129]
**IRE1a**	IRE1a negatively regulates BMP2-induced osteoblast differentiation through UPR and ER stress pathway	[173]
**PERK**	PERK promotes intestinal stem cell proliferation in response to ER stress and limits drosophila lifespan	[118]
**RCAD/Ufl1**	RCAD/Ufl1 is essential for hematopoietic stem cell function and murine hematopoiesis associated with ER stress and UPR signaling pathway	[81]
**Runx1**	Runx1-deficient HSPCs leads to lower p53 levels, reduced apoptosis, an attenuated unfolded protein response, and accordingly are resistant to genotoxic and ER stress	[72]
**SEL1L**	Downregulation of SEL1L synergy enhances VPA cytotoxic effects to GSCs proliferation and self-renewal properties through UPR and ER stress	[133]
**SMN1**	Inhibition of ER stress improves survival of SMA patient-derived induced pluripotent stem cells produced motor neurons	[200]
**TAU**	FTD neurons shows an activation of the unfolded protein response, links to mutant TAU protein	[103]
**UFBP1**	UFBP1 deficiency leads to elevated ER stress and initiation of unfolded protein response (UPR), following causes cell death of hematopoietic stem/progenitor cells	[71]
**Wfs1**	Wfs1 might protect cells during neural differentiation via regulating UPR response in adult brain	[53]
**XBP1**	The transcription factor XBP1 is selectively required for eosinophil differentiation without inducing parallel endoplasmic reticulum (ER) stress signaling pathways	[83]
**XBP1**	ER stress transcription factor Xbp1 directs intestinal stem cells and suppresses intestinal tumorigenesis through restraint of a pathway that involves an Ire1α- and Stat3-mediated regenerative response of the epithelium	[117]
